# Efficacy of Single-Channel EEG: A Propitious Approach for In-home Sleep Monitoring

**DOI:** 10.3389/fpubh.2022.839838

**Published:** 2022-04-12

**Authors:** B. L. Radhakrishnan, E. Kirubakaran, Immanuel Johnraja Jebadurai, A. Immanuel Selvakumar, J. Dinesh Peter

**Affiliations:** ^1^Department of Computer Science and Engineering, Karunya Institute of Technology and Sciences, Karunya Nagar, Coimbatore, India; ^2^Department of Computer Science and Engineering, Grace College of Engineering, HWP Colony, Thoothukudi, India; ^3^Department of Electrical and Electronics Engineering, Karunya Institute of Technology and Sciences, Coimbatore, India

**Keywords:** sleep, sleep disorders, sleep monitoring, remote sleep monitoring, sleep quality, sleep stage classification, single-channel EEG

## 1. Introduction

Sleep is a pivotal biological process and has generally been accepted as a critical factor in human health. Even though the whole function of sleep is not very well studied, it is associated with physical and mental wellness (1, 2). The transient disruptions in regular sleep patterns known as acute sleep deprivation impair cognitive skills. In contrast, long-term sleep abnormalities such as chronic sleep deprivation are related to disease development (3, 4).

Poor sleep quality (5) (as outlined by National Sleep Foundation recommended parameters sleep latency, wake after sleep onset, number of >5 minutes awakenings, and sleep efficiency) has an association with a slew of major medical conditions ranging from obesity and diabetes to neuropsychiatric disorders (6–8). Recent research findings also reveal the associations of poor sleep quality with cardiometabolic risk (9), diabetes (7), weight gain (6), impaired appetite (10), cognitive decline (11), mood changes (12), depression (13), immune function (14), and cancer (15). Global sleep trends indicate that average sleep time is diminishing (16, 17). Besides, sleep-related disorders are on the rise (18, 19). Considering these trends and the importance of sleep for health, a better understanding of sleep characteristics is a public health goal (20, 21).

Polysomnography (PSG) is the gold standard for objective sleep physiology evaluation and has proven to be the most helpful tool for diagnosing sleep-related breathing disorders such as obstructive sleep apnoea, central apnoea, hypopnea, and other respiratory disorders, and also for screening less common sleep disorders, including neurological disorders, such as narcolepsy, parasomnias and seizure disorders, restless legs syndrome and periodic limb movement disorder, depression with insomnia, and circadian rhythm sleep disorder in-clinic sleep assessment and sleep disturbances treatment (22, 23). A PSG study records distinct physiological signals, including electroencephalogram (EEG), electrooculogram (EOG), electromyography (EMG), electrocardiogram (ECG), respiration, pulse oximetry, and other parameters (24). After acquiring the PSG recordings, it is converted into 30-s epochs, and each epoch is mapped to a particular sleep stage (such as N1, N2, SWS, W, REM) manually by a sleep expert or technician, termed as sleep scoring (sleep staging/sleep stage classification). Traditionally more than one technician is involved in this process to avoid biases in marking sleep stages. The accuracy of sleep scoring depends on the expertise of the technicians (25). Although PSG use in clinical sleep medicine has significant benefits, the high cost is a barrier to its accessibility to many populations. Moreover, while undergoing an overnight PSG test, factors such as the unfamiliar sleeping environment with limited privacy, skin irritation due to electrode adhesion, and the numerous leads attached to the person, could obstruct sleep, undermining the accuracy of the recordings (26). Recent advancements in technology play an indispensable role in developing reliable portable monitors (PMs) that support the evaluation of sleep in-home and assist in overcoming the limitations of in-clinic PSG assessment (27). In general PMs are categorised as type 2 (at least seven channels), type 3 (minimum of four channels) and type 4 (either one or two channels) (28).

After considering the underpinning proof of a connection between sleep and wellness, challenges in the traditional sleep evaluation, the need for quality data for the sleep clinicians, this article discusses the significant importance of single-channel EEG in home-based sleep monitoring and analysis. Besides, to further explore the opportunities behind single-channel EEG this article highlights the need for in-home sleep monitoring and its challenges and the role of AI in sleep monitoring. Finally, the challenges in the presented technique and opportunities for future research are presented.

## 2. In-home Sleep Monitoring

In-home sleep monitoring is gaining popularity because of its convenience, non-invasive, and self-administrable. In-home sleep monitoring usually uses type 4 sleep monitoring devices. These devices are either picked up by the patients from the clinic or delivered to their homes (29, 30).

### 2.1. Type 4 Sleep Monitoring

A wearable sleep monitoring system (type 4) is widely used in-home sleep monitoring; these systems detect sleep stages based on any one or two of the signals such as brain waves, heart rate, pulse rate, respiration rate, movements, and other types of signals (body temperature, snoring, etc.) Even though different signals are used for sleep stage analysis, brain signals(EEG) provide more accuracy in specific sleep stage detection and analysis (27). For sleep professionals looking for a more trustworthy, long-term documented total sleep time evaluation, single-channel EEG could be a helpful tool (31). Affordable medical-grade EEG on a large scale is conceived by reducing the electrodes, making it more comfortable, miniaturised, and low-cost. Neurosky (single channel system), Muse and Melon (3-4 electrodes), iBrain, Zeo, and Ear-EEG are consumer-grade products available in the market (32–34).

### 2.2. COVID and Sleep

Due to the COVID-19 pandemic, people are staying indoors; sleep habits have changed. Sleep disorders are common during the COVID-19 pandemic, affecting roughly 40% of the public and healthcare population (35, 36). Furthermore, non-emergency health services like sleep labs were shut to reduce the infection rate (37). During the pandemic, there has been a significant increase in the usage of PMs for sleep monitoring. Most healthcare providers are expecting this trend to continue in the future (38).

### 2.3. Need for In-home Sleep Monitoring

Sleep must be tracked in a free-living setting and in an unobtrusive manner to ensure that the sleep captured is as representative of regular sleep as feasible in order to understand the role of sleep in health and disease. Due to the limitations of PSG, most people are only monitored for a single night. Monitoring during one night, on the other hand, is insufficient to ascertain the actual sleep condition. Long-term, at-home monitoring is required to optimise effectiveness and obtain proper follow-up (29). There are currently various options available for sleep monitoring outside the laboratory using type 4 PM devices (39). Compared to the traditional PSG, the single-channel scheme will save money (40) and make data collection much more straightforward, and valuable in a situation like the COVID-19 pandemic (41, 42). A comparison work done by Lucey et al. (43) show that single-channel EEG can assess REM, combined Stages N2 and N3 sleep, and a variety of other indicators, including frontal slow-wave activity, in a way that is equivalent to polysomnography. Therefore, single-channel EEG based PMs can serve as a better alternative to traditional sleep monitoring, and a better solution for in-home monitoring (44). The significant advantage of in-home sleep monitoring is convenience, level of comfort, and less expensive (45).

### 2.4. In-home Sleep Monitoring Challenges

A home sleep study records your breathing patterns while you sleep in your own bed using small, portable monitoring equipment (30). Even though in-home sleep study is convenient, comfortable, easily accessible and less expensive, it is having a set of challenges listed below.

#### 2.4.1. End User Requirements and Acceptance

An excellent in-home sleep monitoring system design must consider the way the sensor is connected to the body and its attachment method. Users may not prefer the inconvenient method (using adhesive) of connecting sensors to the body and connecting too many sensors. Hence, striking a better balance between user's requirements and acceptance is necessary to design an in-home sleep monitoring device (30, 46).

#### 2.4.2. Long-Term Monitoring

The capacity to do prolonged monitoring is essential for an effective sleep monitoring system. For reliable results and early detection of aberrant sleep abnormalities, long-term monitoring is required. In order to accomplish this, sleep monitoring devices should be low-cost, simple-to-use, and easily accessible (46). The capacity to do prolonged monitoring is essential for an effective sleep monitoring system. For reliable results and early detection of aberrant sleep abnormalities, long-term monitoring is required. In order to accomplish this, sleep monitoring devices should be low-cost, simple-to-use, and easily accessible. To this end, a bed based sensor (47), ear-based EEG (48), Wireless polysomnography system based on the Internet of Things (49), and posture recognition based sleep monitoring (50) proposed.

#### 2.4.3. Seamless Data Sharing With Healthcare Providers

Sleep can be monitored using wearables for a wide range of physical and mental disorders. The majority of the studies relied on commercially accessible gadgets that are linked to smartphones or tablets (34, 51). Wearables can be used to track sleep data. Sleep data can be collected and sent over the internet to a remote clinical on-premise server or cloud server for further analysis, evaluation, decision-making, and treatment. In the captured data, applying machine or deep learning to evolving trends, and instantly alerting patients, nurses, and physicians is a powerful ability (46). Sharing data from a remote location is still a challenging task due to connectivity issues (52).

#### 2.4.4. Data Privacy Issues

Long-term sleep data collection is more comprehensive and diverse. There is an increased chance of user personal information being leaked. Even though, informed consent is used before data collection; given the great value and growing popularity of big data apps, data sharing privacy is a big concern (29). Blockchain technology has the potential to address the issue of privacy and secure data sharing. Implementation of blockchain in real-life applications are in the inception stage and more understanding is needed (53).

## 3. Role of AI in Sleep Stage Classification

Sleep technicians must verify each epoch manually to perform the sleep scoring, and it has limitations such as labour-intensive and time-consuming and inter-rater variability (25). Kappa (κ) measures the manual sleep scoring performance to estimate interrater reliability, representing an agreement between epoch-to-epoch. The benchmark κ value against human-AI algorithm agreement is 0.68–0.76 approximately (54). Analyzing hours of patient sleep records is not easy due to the aforementioned limitations. Therefore, researchers extensively use machine-learning (ML) and deep-learning (DL) techniques to score the sleep stages from the sleep data automatically (55).

ML models' performance depends on the representative features extracted from the EEG signals. The features are either extracted from 30-s data (epoch) or sub-bands of decomposed signals. Specific studies have extracted features from both the 30-s epochs as well as sub-bands or only from the decomposed signals. The commonly used EEG signal decomposition techniques used in the literature include EMD (empirical-mode decomposition), EEMD (ensemble-empirical-mode decomposition), wavelet-based, FFT (fast Fourier-transform), and frequency such as alpha, gamma, etc., based decomposition (56–58). Once the features are extracted from the EEG signal, it is given as an input (training/test data) to the ML model. Sleep scoring is a multi-class classification problem (mapping five/six sleep stages based on input features). Researchers have tested multiple ML models for automated sleep scoring systems *viz*.: tree-based models (Random forest, SVM, XGBoost, etc.), clustering (KNN), and an amalgamation of distinct models known as ensemble learning (stacking, boosting, bagging, and blending). These approaches achieved 74.5 to 91.9 % accuracy levels for a standard five-class classification (57, 59–63).

**Figure 1:**
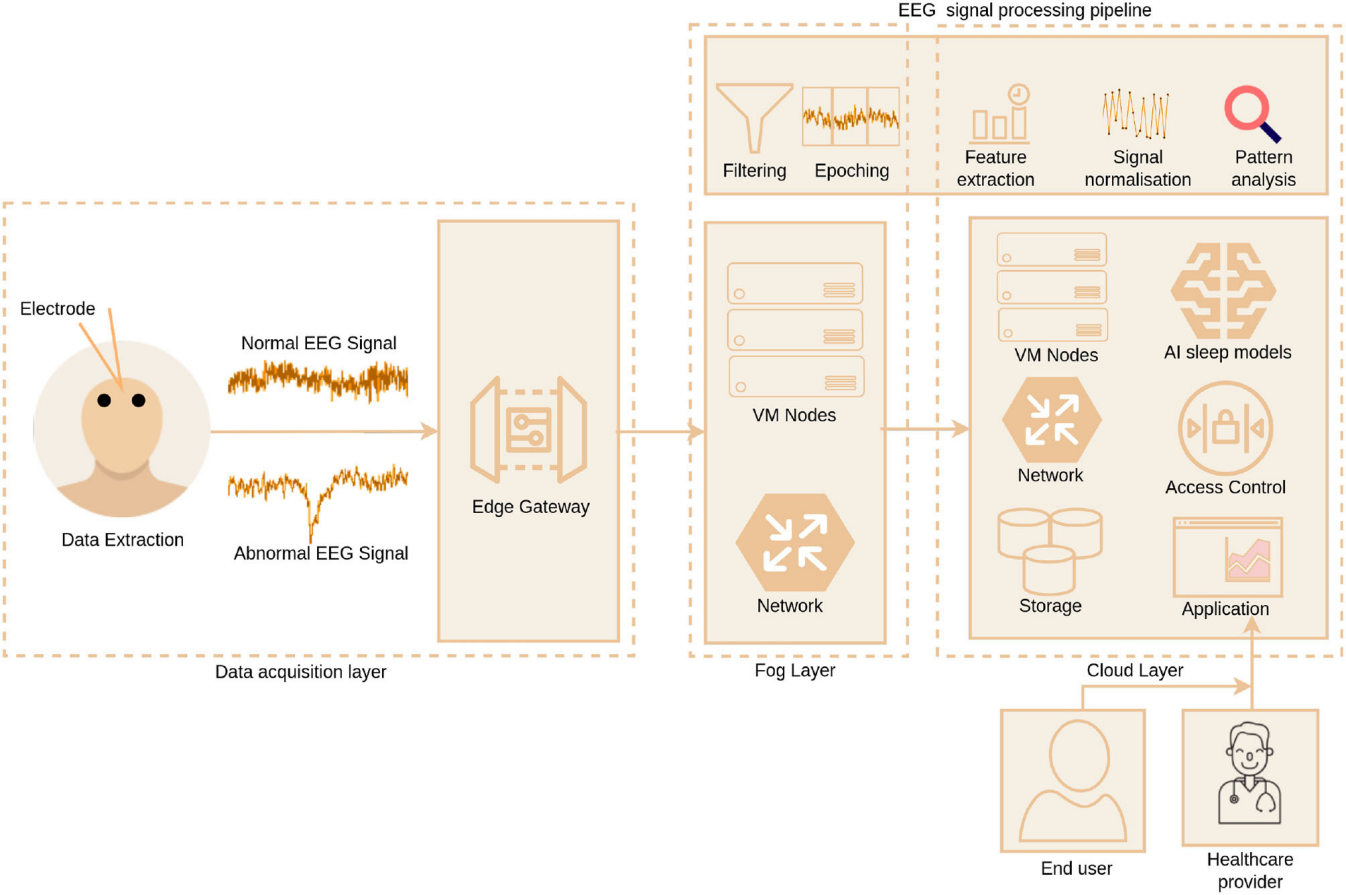
Overview of sleep data extraction and analysis using cloud computing.

Data dimensionality is a notable concern in ML-based systems when dealing with high data-dimension like PSG, which overfit the ML model. Besides, ML-based sleep scoring systems have distinct phases, such as feature extraction, selection, and classification that run as separate tasks. Recent improvements in ML facilitate ways to run discrete tasks together. DL models provide encouraging results in sleep scoring using single-channel EEG based systems (64). The literature's proposed DL-based sleep scoring systems generate features from the input sequence or employ the manually extracted features. A few existing works have used 90- and 60-s epochs instead of traditional 30-second epochs for classification in order to represent the temporal relations between the sleep stages and enhance the sleep scoring results (65). Authors have explored many individual and cascaded DL models for automated sleep scoring systems. Some of the recently proposed DL based works are: convolutional neural network (CNN) (65, 66), IITNet, a CNN and Recurrent Neural Networks (RNN) based network (67), SingleChannelNet (SCNet), a CNN based model. The DL based approaches achieved 83.9 to 92.9% for a standard five-class classification (68), SleepStageNet (69), Long short-term memory (LSTM)-RNN (70). The Figure 1 illustrates the overview of sleep data extraction and analysis pipeline using cloud computing. The EEG signals are extracted from the individuals suspected of sleep-related issues. Then the signals are filtered to remove the noise and converted into 30-s epochs for identifying the specific sleep stages. Next, features are extracted manually using the methods mentioned above or fed the signals to the DL model for automatic feature extraction. The trained model is used to detect sleep stages automatically.

## 4. Role of Cloud in Sleep Monitoring

Remote health monitoring is one of the key benefits of the digital era; it allows for remote tracking and monitoring of an individual's health-related factors, as well as sharing with healthcare specialists (71). Recent advancements in sensors, microcontrollers, and communication systems have made significant advances in remote healthcare, allowing for collecting health data from individuals. The Internet of Things (IoT) has pushed the healthcare industry to adopt completely digitised e-health systems and will undoubtedly play a role in remote healthcare (72). The application of wearable sensors to assess an individual's health or well-being status is most attractive in out-of-hospital circumstances (73).The IoT and Cloud integration is gaining traction in digital healthcare and is used for digital sleep monitoring and assessment. The modern digital sleep health applications comprise data acquisition, fog, and cloud layers depicted in Figure 1. The data acquisition layer uses wearable EEG devices for EEG data acquisition. The fog layer brings the cloud to the end-user devices to perform basic preprocessing of data and initial assessment at the user end. Also, It supplies the homogeneous data to the sleep application running in the cloud that simplifies the processing. The cloud layer provides the compute and storage for the applications. The data acquired by the wearable EEG devices needs to be accumulated and kept safely for future reference, trend analysis and retraining the deployed models. Cloud storage is the viable option to store the sleep data since it offers cost-efficiency, secure sharing, synchronisation and scalability. Healthcare providers leverage ML or DL models using the EEG data stored in the cloud to perform automated sleep stage analysis, resulting in more accurate insightful findings, visualisation, and diagnosis. Analysing EEG data and running sleep stage predictive models support real-time decision making. Despite the merits of the cloud, there are specific challenges when connecting EEG devices in real-time and storing data to the cloud. In the fog layer, EEG data may miss due to improper wearing of the device or malfunction of the sensor. Cloud layer stores and shares EEG data among applications; however, there is growing concern concerning privacy, security and data access (74–76).

## 5. Discussion

In a technology-driven world, sleep is usually the first thing individuals compromise when they feel pressed against time. Chronic sleep deficiency is associated with cognitive skills, as well as health consequences, such as obesity, diabetes, neuropsychiatric disorders, cardiometabolic risk, impaired appetite, mood changes, depression, immune function, and cancer (6–10, 13–15). COVID-19 pandemic phenomenon increased the sleep-related issues and inaccessibility to sleep clinics. Despite continuous sleep monitoring having its benefits, COVID-19 highlighted its importance (35, 36). Contemporary advancements in sensing techniques, data analytics, and AI systems allow sleep monitoring ubiquitously and unobtrusively (38, 54, 65). Sleep monitoring research using single-channel EEG gains an excess of attraction and momentum since it supports continuous monitoring non-obstructive, aids detecting specific sleep stages accurately and is easy to employ at home (44, 45).

Many ML and DL-based models proposed in the literature achieved better accuracy and kappa (κ) values. Although ASSC systems produce better results, a set of specific challenges exist, such as database variability, channel mismatch, class imbalance, inter-class distinction, computational complexity, and scoring issues (63, 65). Most of the ML and DL-based models proposed in the literature used distinct datasets; these data had been collected from different individuals. Hence, this creates a bias during the comparison of results, and there is a need to investigate the robustness of the model (65). There has been a considerable interest among researchers to design sound in-home single-channel EEG-based sleep monitoring systems. Channel mismatch is another significant factor that hampers the performance of portable ASSC systems (65).

Effectively managing class imbalance (among stages of sleep) is an obligation of an ASSC system. All PSG recordings from healthy persons are generally imbalanced due to the less representation of the S1 stage. Consequently, both ML and DL models render limited performance in classifying the N1 stage (65). To handle this issue, researchers took different approaches. Zhou et al. (62) proposed a method that adjusts the class weights to achieve class balancing, and it has significantly improved the N1 stage recognition to 72.52%. Another author Jiang et al. (58) balanced only the training dataset to make all the classes equal. This approach improved the S1 stage detection from 0.44 to 0.58 (recall score). Moreover, this approach slightly improved the overall detection of less represented classes. In Sors et al. (66) class balancing is done using cost-sensitive learning. That improves N1 and N3 stage results significantly.

Sleep is referred to as a continuous event, and there is no clear-cut boundary between sleep stages. Therefore, different sleep stages, particularly transitioning stages, are tough to distinguish due to subtle inter-class distinctions (65). The sleep stages N1and N2 have similar features. Similarly, N1 and REM also have similar features. This similarity confuses the classifier and even human experts. Moreover, the S1 stage is the transitional stage between W and REM. Hence, among the tested classifiers in literature, N1 stage detection is still challenging (58). Some studies have combined the N1 and N2 stages as light sleep (LS), improving detection accuracy. This limitation can be further improved by incorporating EMG or EOG signals (69). The study by Michielli et al. (70) used a multi-class (N1 and REM combined) and binary class (N1 and REM) approach, this approach improving the N1 stage detection considerably. The performance of the ASSC systems depends on the ML/DL model's complexity. Hence, it is necessary to strike a balance between model complexity and performance (65). Training neural networks like RNN on GPU have tight memory size. This limitation is managed using lesser training sequences.

An in-home ASSC system based on single-channel EEG is required to alleviate the problems in manual scoring and enable the development of a convenient, comfortable, and less expensive in-home sleep monitoring system (63). Single-channel systems have excellent scope in terms of convenience and cost-effectiveness. When comparing the results of recent studies, single-channel EEG shows significant performance. However, there are fewer number studies that validate the results against PSG. Conducting more validation studies among the diverse group (including normal and person with a sleep disorder) using single-channel EEG and validating against PSG would improve the reliability and validity of the ASSC systems. A cloud-based trained and tested framework is necessary to provide accurate multi-model sleep scoring and analysis, seamless data sharing, and facilitate connecting health providers. The importance of continuous monitoring is evident in the literature. Continuous monitoring accumulates more data, detects sleep anomalies, and predicts health-related consequences. Cloud providers offer HIPPA compliance cloud storage that supports securely storing and sharing data. All the services offered by the cloud providers may not be HIPPA compliant. Therefore, before adopting a cloud service, it is essential to verify its HIPPA compliance. Convenience and accuracy is the prime objective of single-channel EEG systems. The missing data issue in the fog layer can be addressed by various methods such as tensor factorization (77). Some studies suggested that adding EMG or EOG sensors may improve the discrepancies in detecting accuracy among N1, N2, and REM stages. However, it causes inconvenience and obstructs regular sleep. Therefore, future research could incorporate heart rate and body movement signals (smartwatches or wearables) with single-channel EEG to improve sleep scoring.

## Author Contributions

BR, EK, IJ, AS, and JP contributed to the study conception and design, literature review, interpretation, and manuscript preparation. All authors contributed to the article and approved the submitted version.

## Conflict of Interest

The authors declare that the research was conducted in the absence of any commercial or financial relationships that could be construed as a potential conflict of interest.

## Publisher's Note

All claims expressed in this article are solely those of the authors and do not necessarily represent those of their affiliated organizations, or those of the publisher, the editors and the reviewers. Any product that may be evaluated in this article, or claim that may be made by its manufacturer, is not guaranteed or endorsed by the publisher.
